# Rhino-Orbital Mucormycosis Presenting As Orbital Apex Syndrome With Central Retinal Artery Occlusion: A Rare Phenomenon

**DOI:** 10.7759/cureus.73400

**Published:** 2024-11-10

**Authors:** Venushia Chandran, Choo Swee Ying, Sharifah Intan Hosnaliza, Rona A Nasaruddin, Jemaima Che Hamzah

**Affiliations:** 1 Ophthalmology, Hospital Kuala Lumpur, Ministry of Health Malaysia, Kuala Lumpur, MYS; 2 Ophthalmology, Faculty of Medicine, Universiti Kebangsaan Malaysia, Kuala Lumpur, MYS

**Keywords:** central retinal artery occlusion, complete ophthalmoplegia, corticosteroids, covid-19, diabetes type 2, orbital apex syndrome, orbital mucormycosis

## Abstract

Rhino-orbital-cerebral mucormycosis (ROCM) is an opportunistic infection that has increased due to COVID-19 with the use of corticosteroids and diabetes being the most important predisposing factors. Orbital apex syndrome with central retinal artery occlusion secondary to mucormycosis is relatively rare. This case report highlights a case of a 62-year-old female with poorly controlled diabetes and a history of COVID-19 two weeks prior, who presented with acute right eye painful visual loss for three days associated with bulging of the right eye and drooping of the eyelid. On presentation, she had no light perception in her right eye, total ocular movement restriction, lid swelling, bulging of the eye, and complete ptosis. Examination of the right eye showed mild conjunctival redness with evidence of central retinal artery occlusion. Contrast-enhanced computed tomography of the brain and orbit revealed evidence of orbital cellulitis with sinusitis. Nasal endoscopy revealed features of fungal sinusitis. Despite multiple attempts of debridement and intravenous Amphotericin B, the patient’s condition progressed and required right orbital exenteration. This report aims to highlight the necessity of high suspicion of ROCM in COVID-19 patients with diabetes, a history of steroid use, and the need to be followed up beyond recovery. Multidisciplinary team management is needed to detect red flag symptoms and signs, diagnose promptly with appropriate microbiological and radiological investigations, and initiate early treatment with antifungal and aggressive surgical debridement for a successful outcome and to prevent the need for extensive surgical measures like orbital exenteration.

## Introduction

Mucormycosis is a lethal and invasive fungal infection caused by members of the Mucoraceae family [1]. Prior to the COVID-19 pandemic, the occurrence of mucormycosis was rarely reported. The prevalence across the globe varied from 0.005 to 1.7 per million population. It was mostly confined to the Indian subcontinent with a prevalence nearly 80 times higher (0.14 per 1,000) and reported predominantly in immunocompromised individuals [2]. However, the COVID-19 pandemic has brought a surge in the number of mucormycosis cases evidenced by the study by Ozbek et al. reporting 958 cases across 45 countries [3] and another study by Sen et al. highlighting 2,826 cases reported in India alone from January 1, 2020 to May 26, 2021 due to use of steroids, coupled with poorly controlled diabetes [4]. Despite timely surgical and medical treatments, the mortality rate remains as high as 46% [2]. Due to its non-specific presentation, there may be a delay in the diagnosis of rhino-orbital-cerebral mucormycosis (ROCM) and subsequently its treatment. As such, clinicians must have a high index of suspicion in all patients with COVID-19 especially those treated with steroids and with other risk factors. This report aims to highlight a patient who had an atypical presentation of orbital apex syndrome with central retinal artery occlusion (CRAO) secondary to ROCM and survived the deadly disease.

## Case presentation

A 62-year-old female with poorly controlled diabetes, hypertension, and a history of treated cervical cancer presented with a sudden onset of painful visual loss in the right eye for three days associated with right eye proptosis, redness, and ptosis. The patient denied any history of recent ocular trauma but revealed that she had a history of COVID-19 infection two weeks prior and was admitted for oxygen therapy and was on intravenous (IV) Dexamethasone 8 milligrams, thrice a day, for three days. She made a good recovery and had no visual complaints during her admission for COVID-19.

**Figure 1:**
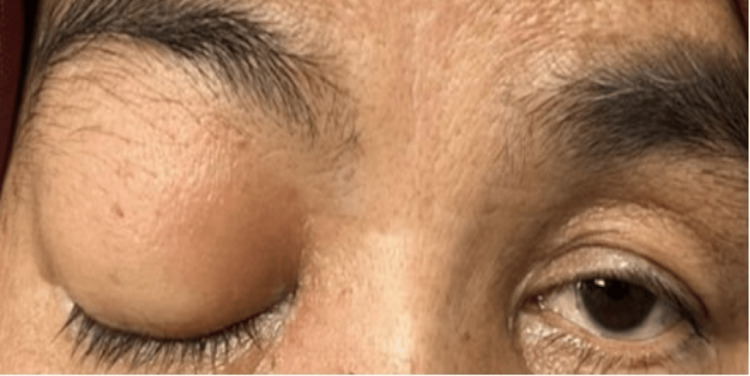
Right complete ptosis showing oculomotor nerve (CN III) palsy that demonstrates the spread of infection and involvement up to at least the orbital apex.

**Figure 2:**
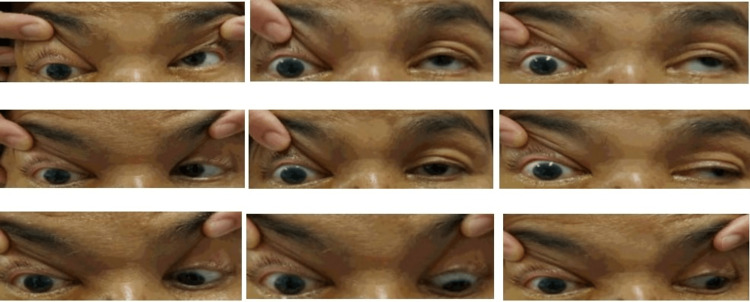
Nine gaze images showing a right “frozen eye” that has no movement in all directions of gazes. This supports the involvement of oculomotor nerve (CN III), trochlear nerve (CN IV) and abducens nerve (CN VI).

**Figure 3:**
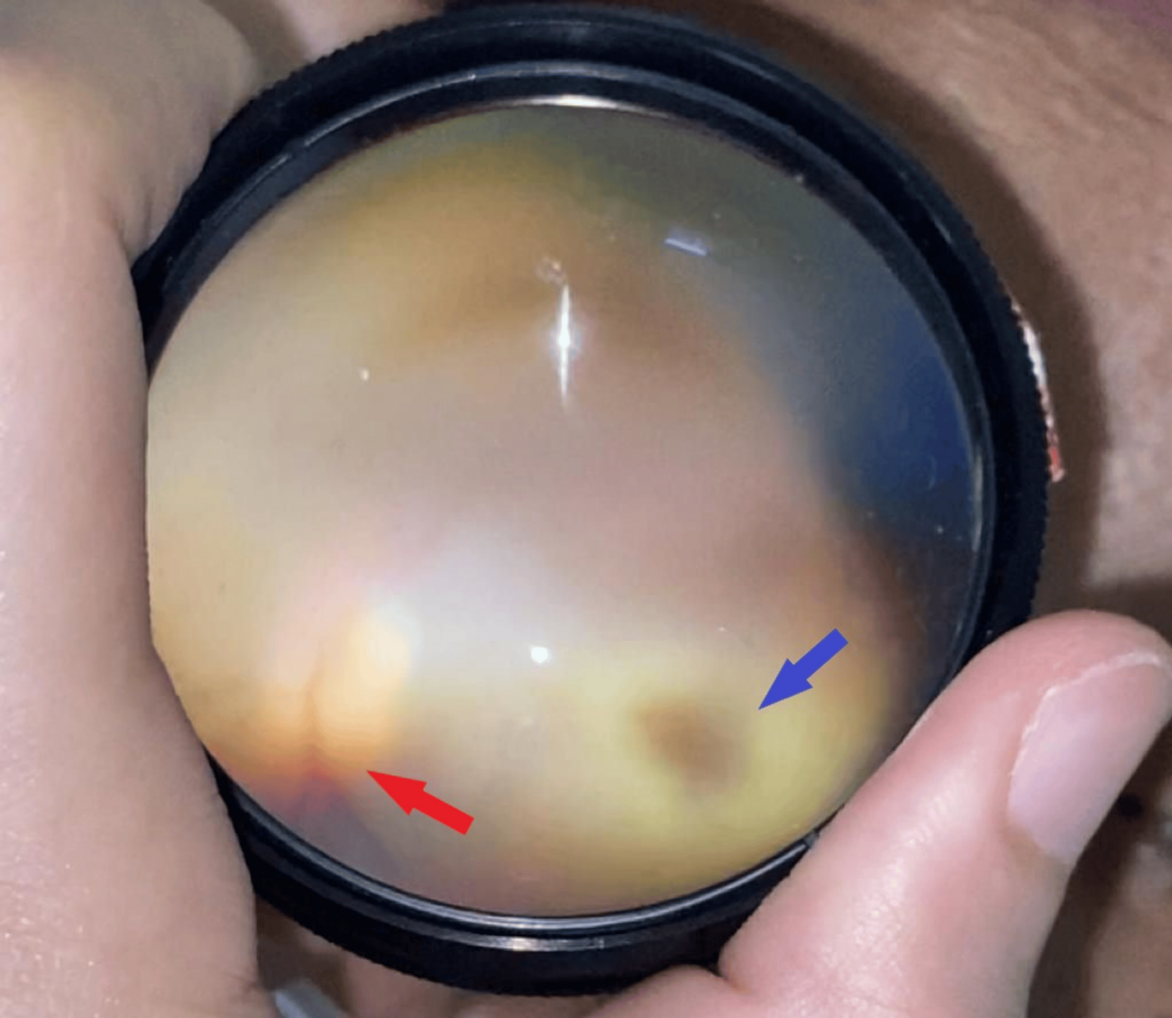
Right eye fundus seen using a binocular indirect ophthalmoscope showing swollen optic disc (red arrow), pale ischemic retina surrounding a pink macula that was seen as a cherry red spot (blue arrow).

On presentation, she had no light perception in her right eye and a visual acuity of 6/12 in her left eye. The right eye relative afferent pupillary defect (RAPD) was positive with lid edema, proptosis, complete ptosis (Figure 1), and total ophthalmoplegia (Figure 2). Examination of the right eye showed mild conjunctival redness with inferior chemosis. The pupil was 5mm, round and sluggish. Fundus examination of the right eye revealed a swollen optic disc with a cherry red spot at the macula surrounded by a pale, ischemic retina (Figure 3), consistent with features of CRAO. Left eye examination showed moderate non-proliferative diabetic retinopathy changes. There was no involvement of other cranial nerves (CN).

The patient’s vital signs were stable, and she was afebrile. However, her glucose reading was high at 16.0 mmol/L (normal range 3-6.1 mmol/L) despite the patient’s normal dose of insulin. Basic blood investigations showed a high white blood cell count of 21.98 x 10^9^/L (normal range 4.0-10.0 x 10^9^/L) with neutrophil predominance. Urgent contrast-enhanced computed tomography of the brain and orbit was done, and it revealed evidence of orbital cellulitis with sinusitis involving the maxillary and ethmoidal sinuses. She was started on IV Ceftriaxone for the treatment of orbital cellulitis and was referred to the Ear, Nose, and Throat (ENT) team for assessment. A nasal endoscopy on day 3 of admission revealed features of invasive fungal sinusitis with eschar in the right nasal mucosa and right maxillary sinus. Thus, IV Amphotericin B was started for the treatment of fungal sinusitis and IV Ceftriaxone was stopped after a week. The patient underwent multiple sessions of endoscopic debridement and fungal deloading within the first 10 days of admission. The nasal mucosa biopsy tissue culture and sensitivity results grew Rhizopus sp. The diagnosis was then revised to rhino-orbital mucormycosis and multidisciplinary team management involving ENT, ophthalmology, and infectious disease (ID) specialties was commenced. Magnetic resonance imaging (MRI) of the brain, orbit, and paranasal sinuses was done two weeks after admission and multiple endoscopic debridement sessions by ENT to assess the extension of fungal infection to determine and guide further debridements. The MRI showed invasive sinusitis with extension into the right pterygopalatine fossa but without intraorbital or intracranial involvement. This led to another session of aggressive debridement up to the pterygopalatine fossa.

**Figure 4:**
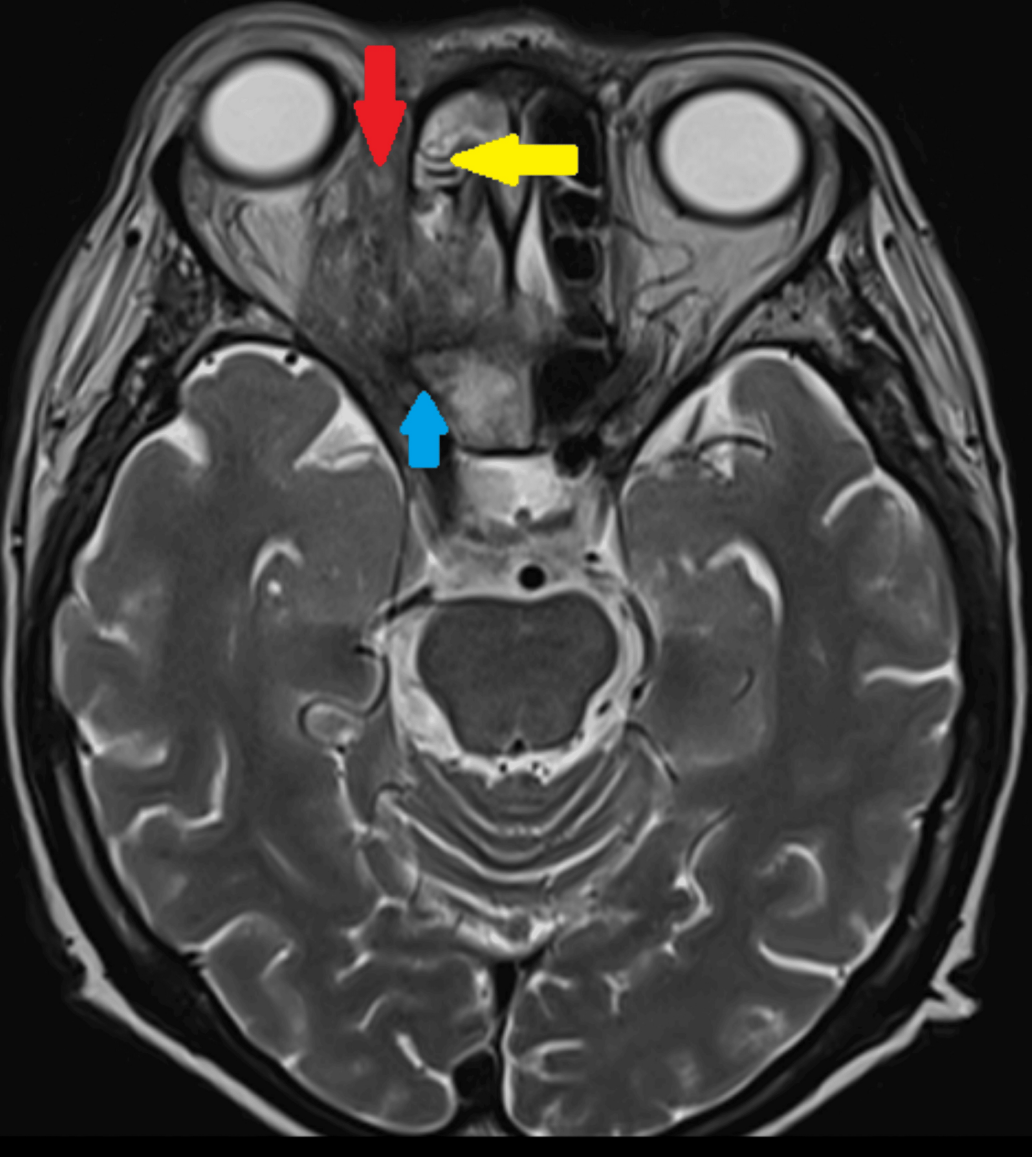
MRI showing hypointense lesion in right ethmoid sinus (yellow arrow) with right eye proptosis and subperiosteal collection at the medial wall of right orbit extending into extraconal compartment (red arrow). Enhancement surrounding optic nerve with extension into orbital apex with dural enhancement of anteroinferior part of right cavernous sinus (blue arrow).

**Figure 5:**
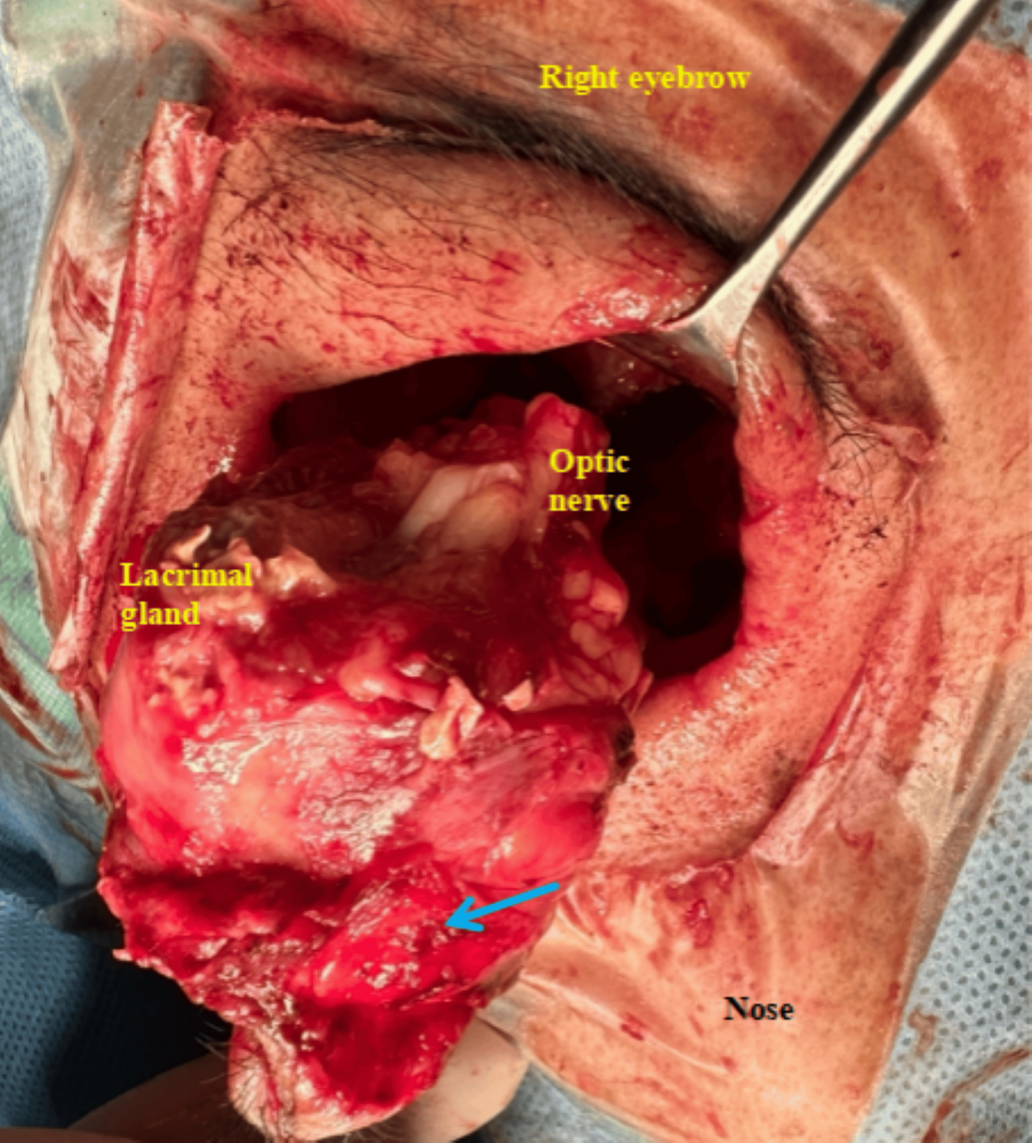
Intraoperative image of exenterated orbital content showing friable necrotic mass medially (blue arrow) at the foci of infection.

**Figure 6:**
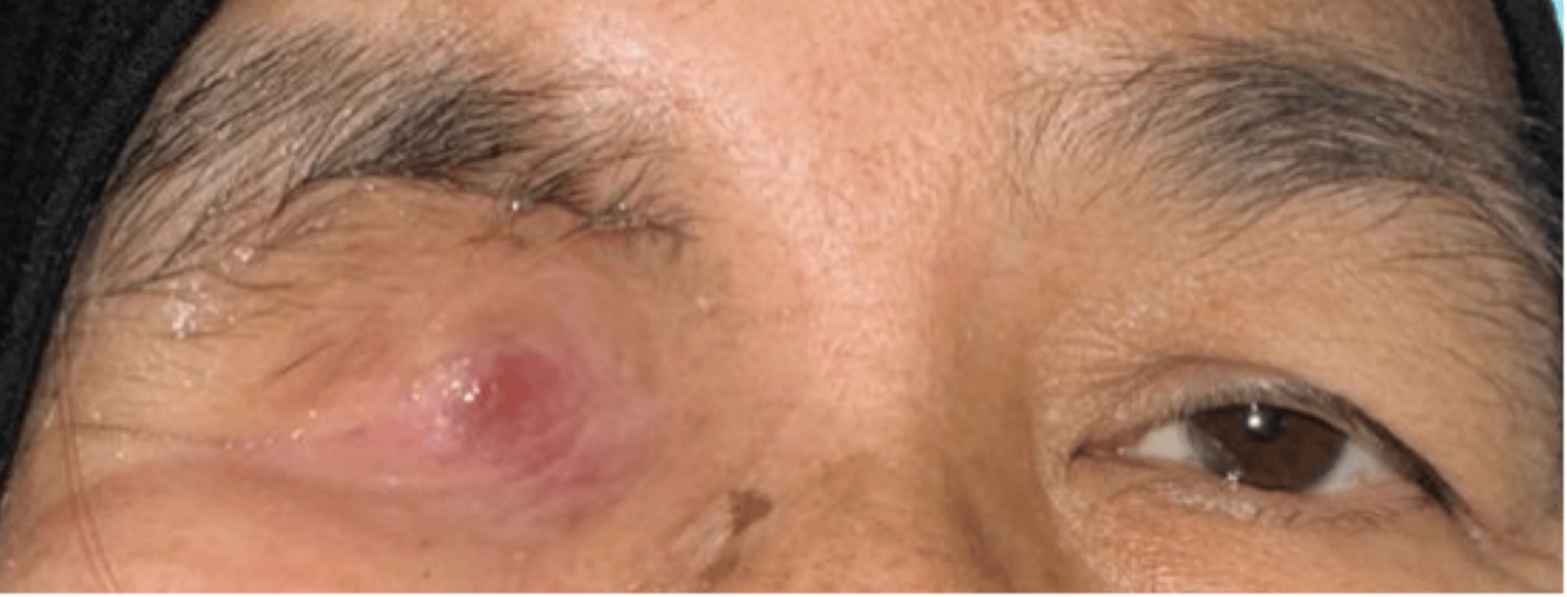
Patient at one year post exenteration of right eye with a healthy well healed wound and no evidence of recurrence of infection.

Despite intensive multidisciplinary care, the patient deteriorated in the ward due to the progression of infection as evidenced by the repeat MRI one month later that revealed a new right subperiosteal abscess with intraorbital extension from the medial wall and dural enhancement of anteroinferior part of cavernous sinus (Figure 4). A multidisciplinary team meeting was held and right subtotal orbital exenteration with lid sparing was decided on after counselling the patient and family. Surgery was performed within a week. Intraoperatively, there was necrotic material medially at the area close to the subperiosteal collection with friable mass and unhealthy base (Figure 5). With combined multidisciplinary care, the patient made a good recovery and was discharged home with an oral antifungal (Posaconazole) for a duration of six months. Figure 6 shows the patient at one-year post right orbital exenteration with no evidence of recurrence of infection. She is currently undergoing cosmetic rehabilitation and awaiting an ocular prosthesis.

## Discussion

Mucormycosis can infect any organ in all age groups and is generally divided into six types based on the anatomical location: rhino-orbital-cerebral, pulmonary, cutaneous, gastrointestinal, disseminated, and other rare forms (endocarditis, osteomyelitis, pyelonephritis, peritonitis) [5]. ROCM contributes to two-thirds of all cases of mucormycosis making it the most common presentation of mucormycosis [4]. Studies show that *Rhizopus *spp. is the most common type and is responsible for 60% of mucormycosis cases and 90% of ROCM cases [2].

**Figure 7:**
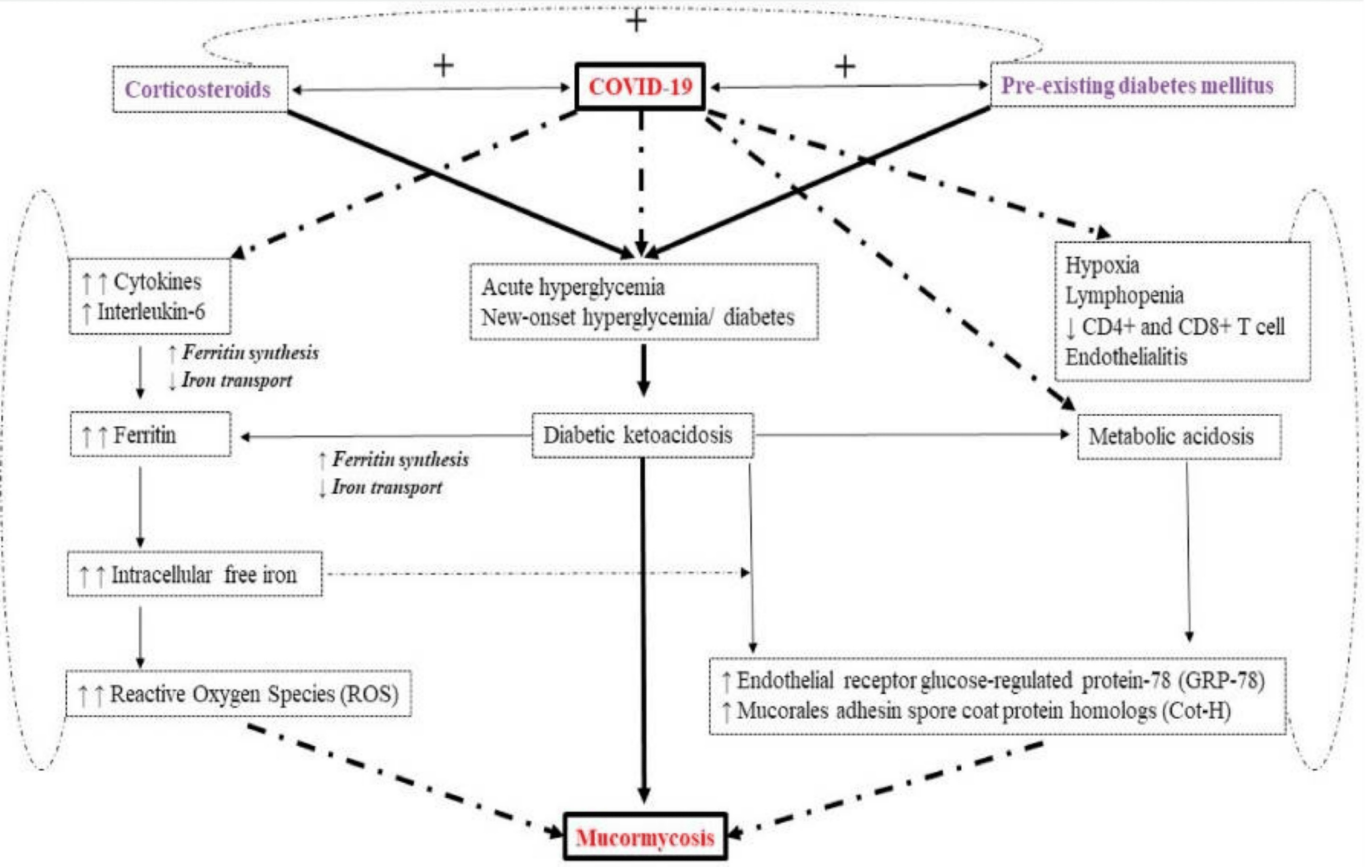
The postulated mechanism for mucormycosis infection in COVID-19 patients. The combination of poorly controlled diabetes, immunosuppressive steroid use, and COVID-19 infection creates a synergistic effect, significantly increasing the risk of mucormycosis through immune dysregulation and favorable growth conditions. Reproduced with permission from Singh AK, et al. [2].

Before the COVID-19 pandemic, mucormycosis rarely affects immunocompetent patients. The at-risk population was those with poorly controlled diabetes mellitus (DM), hematological or solid malignancies, severe neutropenia, primary or acquired immunodeficiencies, and post-transplant patients [4]. The postulated mechanism for COVID-19-associated mucormycosis infection is shown in Figure 7. COVID-19 infection alters a patient’s immune system and causes the cell-mediated immunity pathway to become ineffective. It increases inflammatory cells such as cytokines and interleukins, reduces CD4 and CD8+ T cells, and causes lymphopenia, endothelialitis, endothelial damage, and thrombosis leading to opportunistic fungal infection [2,5]. Concomitant poorly controlled diabetes with or without diabetic ketoacidosis creates a hyperglycemic and acidotic environment that is conducive to the growth of the fungus. Patients with systemic acidosis have high levels of available serum iron due to proton displacement causing ferric iron (Fe3+) to be reduced to ferrous iron (Fe2+) at the cell surface of Mucorales. This is then fueled upon by the Mucorales for growth; further encouraging their rapid spread [5]. The combined effect of high glucose and iron levels will induce the expression of glucose-regulated protein 78 (GRP78) which causes the mucorales to invade and damage human endothelial cells [6]. The use of corticosteroids as a part of the COVID-19 treatment regime also further suppresses the immune system and contributes to hyperglycemia, rendering a more favorable environment for the invasion and growth of the mucorales. It was found that a cumulative dose of higher than 600mg for prednisolone and 2-7g of methylprednisolone increases the risk of immunocompromised patients developing mucormycosis [2]. In this patient, her preexisting poorly controlled diabetes, history of cancer, and the recent COVID-19 infection coupled with the systemic short course of IV corticosteroids had led to further suppression of her immunity enabling rapid spread of the infection.

According to a study done by Sen et al., the mean interval of onset of ROCM symptoms from the diagnosis of COVID-19 was 14.5 days with 56% of the 2286 patients developing within 14 days and 44% after 14 days and all within 90 days from diagnosis of COVID-19 [4]. Symptoms on presentation vary with the stage of ROCM disease. Fungal invasion in ROCM first occurs by inhalation of the fungal spores that get deposited on the nasal turbinate and invade the nasal mucosa. It progresses to most commonly the ethmoidal and maxillary sinuses followed by the frontal and sphenoid sinuses [7]. From paranasal sinuses, it can spread into the orbit by eroding the medial orbital wall and once in the orbit, it can commonly manifest itself as CRAO, orbital apex syndrome, endophthalmitis/panophthalmitis, and globe erosion. In a study by Srivastava et al., in COVID-19-associated mucormycosis patients, 31 out of 89 (34.8%) had orbital apex syndrome and almost half (15) of the patients had CRAO [8]. CRAO is most commonly found in hypercoagulable conditions; however, in ROCM, it occurs due to predilection of the fungus for internal elastic lamina of blood vessels causing infiltration into the central retinal artery from the orbit. This initiates a phenomenon of necrotizing vasculitis and thrombosis leading to retinal ischemia [9,10]. From the orbital apex, the fungal infection can extend further into the cavernous sinus, internal carotid artery, and ultimately the brain parenchyma which may be life-threatening or cause severe debilitation if not detected and treated early [8].

As mucormycosis is an opportunistic infection, misdiagnosis often occurs leading to late identification and a high mortality rate associated with this condition. Therefore, it is vital to have a high index of clinical suspicion and a low threshold for diagnosis in patients with risk factors who display red flag signs such as nasal discharge, nasal stuffiness, foul smell, facial or periorbital swelling, facial pain or palsy, eye movement restriction, ptosis or drop in vision. Pulmonary physicians and intensive care teams need to be alerted on these clinical symptoms and signs as the majority of COVID-19 patients with moderate-to-severe disease are attended by them. This is to ensure earlier detection and timely referrals to ophthalmology, otorhinolaryngology, and IDs teams as the 30-day mortality rate is doubled with even a delay of six days in treatment initiation [7].

Management of mucormycosis is challenging and requires a multidisciplinary approach which includes reversing underlying risk factors such as hyperglycemia and acidosis, prompt treatment with antifungal therapy, and finally, surgical debridement if the disease is unresponsive to systemic antifungal alone [5]. The most effective antifungals to date are lipid formulations of Amphotericin B, Posaconazole, and Isavuconazole [7]. In limited resources situations, Amphotericin B Deoxycholate or Amphotericin B Lipid Complex may be used although they have lower efficacy and high systemic toxicity [11]. Retrobulbar Amphotericin B is another option of treatment to increase drug penetration to the affected site [11]. Medical therapy may frequently be insufficient to effectively cure patients due to fungal angioinvasion, thrombosis of blood vessels, and extensive tissue necrosis that decreases the drug bioavailability at the site of infection [12]. Patients may have to undergo multiple attempts of surgical debridement and in cases of ocular involvement that is not responding to systemic antifungal or extensive orbital involvement, the patient may be subjected to orbital exenteration to prevent progression of disease. The aim of treatment is in the order lifesaving, control of disease progression, and finally vision saving. In this case, the timely intervention of orbital exenteration halted the disease progression and was lifesaving. Orbital exenteration leads to psychosocial issues due to permanent facial disfigurement and as such thorough multidisciplinary counseling is needed. As the patient was counseled pre-operatively regarding the cosmetic defect and the option of the cosmetic prosthesis, the patient and family were more agreeable and accepting of the surgical intervention. Postoperatively, the patient has to be given step-down treatment and continued with oral antifungal, Posacanazole, or Isavucanazole for a period of three to six months with serial radiological investigation to ensure regression of disease [11]. Our patient showed no recurrence of disease and was able to lead a good life while awaiting her cosmetic prosthesis due to her strong family support.

## Conclusions

ROCM is a relatively challenging disease to diagnose for clinicians as it is an opportunistic infection that may present atypically. Hence, a high index of suspicion is necessary especially in post-COVID-19 patients with risk factors of immunosuppression, presenting with acute blurring of vision associated with features of sinusitis. Patients with risk factors should be monitored for at least three months post-COVID-19 diagnosis to ensure earlier detection if symptoms or signs develop. Patients and family members should be alerted of the red flag symptoms so that medical care is sought early. Prompt diagnosis with relevant investigations and treatment with a multidisciplinary approach is needed to preserve vision, spare patients from lifelong cosmetic blemishes, and avoid life-threatening complications. Standardized guidelines should be developed and implemented to ensure well-rounded and optimal care. Due to the high mortality associated with ROCM, preventative steps must be taken in high-risk populations by early identification and management of the risk factors to ensure that the number of patients who succumb to this illness can be reduced. Low-dose steroids may be used cautiously, and tighter glucose control should be practiced in those with risk factors. COVID-19-associated ROCM needs to be addressed aggressively with a combined effort from the government in providing the logistics and a well-versed multidisciplinary medical team.
